# Molecular mechanism underlying the effect of illumination time on the growth performance of broilers via changes in the intestinal bacterial community

**DOI:** 10.7717/peerj.9638

**Published:** 2020-08-03

**Authors:** Yongfen Wang, Zhen Zhang, Pengkun Yang, Miaorui Zhang, Lei Xi, Qiong Liu, Jingang Li

**Affiliations:** 1 College of Life Sciences, National Engineering Laboratory for Resource Development of Endangered Crude Drugs in Northwest China, Shaanxi Normal University, Xi’an, China; 2 College of Food and Biology Engineering, Henan University of Animal Husbandry and Economy, Zhengzhou, Henan, China; 3 College of Biosystems Engineering and Food Science, Zhejiang University, Hangzhou, China

**Keywords:** Illumination, Microbial community, Melatonin, Insulin, Feed-to-meat ratio

## Abstract

The circadian rhythms associated with light have important effects on the growth, metabolism, immunity and reproduction of broilers. However, there is a lack of systematic evaluations of the effect of the light cycle on intestinal microbes and the nutritional metabolism of these microbes in broilers. This study was designed to study the effects of the light cycle on the intestinal bacterial community structure and growth of broilers. In this study, Arbor Acre (AA) broilers were fed under a short photoperiod (1L:23D), a long photoperiod (23L:1D), and a normal photoperiod (16L:8D), respectively. The feed conversion ratio of the broilers was calculated, and the levels of endocrine hormones, such as melatonin, insulin and glucagon, were determined. Intestinal contents were collected from the small intestines of the broilers after slaughtering, and the V3+V4 region of the 16s rDNA gene was sequenced. The results demonstrated that changes in the light cycle could affect the synthetic rhythms of melatonin, insulin and glucagon. Compared to short and normal photoperiod, long photoperiod significantly increased the abundances of *Barnesiella* species in intestinal microbes and decreased the abundances of *Bacteroides* and *Alistipes* species. Cluster of Orthologous Groups of proteins analysis indicated that prolongation of the illumination increased the abundances of bacterial genes with glycometabolic and membrane transport functions in intestinal microorganisms. A model was established in this study, and our results showed that prolonged illumination altered the intestinal microbial community structures of broilers, increased the absorption and utilization of polysaccharides in broilers, and reduced the feed-to-meat ratios. To the best of our knowledge, this is also the first study to describe the molecular mechanism underlying the effects of the light cycle on the uptake and utilization of nutrients that occur via modification of the intestinal microbial community structure in broilers.

## Introduction

The circadian rhythms of the animal body are closely associated with physiological activities, such as metabolism, hormone secretion, temperature regulation, the cell cycle and feeding (Mohawk, Green & Takahashi, 2012). Light is the most important regulatory factor of circadian rhythms in animals (Van der Vinne et al., 2020).

Regular microbial communities maintain the metabolism and immune stability of the intestinal epithelium and the whole body (Kim et al., 2018; Nicholson et al., 2012). The high feed conversion ratio (FCR) of broilers is closely related to specific internal microbial communities, while the intestinal microbial communities are susceptible to external diet and environmental conditions. The normal microbial communities of the intestine produce various nutritive materials, such as short-chain fatty acids and amino acids, by metabolizing carbohydrates, proteins, fatty acids and other macromolecular nutrients. Due to the extensive interactions among microbes, microbial metabolites and intestinal epithelial tissue, changes in the microbial community structure are often associated with changes in the function of the intestinal environment. Furthermore, changes in the microbial community structure influence metabolic homeostasis in the body (Claus et al., 2011). Intestinal microorganisms regulate lipid uptake by invading cells in the small intestine. The impact of intestinal microorganisms on the host is highly significant when associated with a high-fat, high-sugar diet (Leone et al., 2015; Wang et al., 2017). Therefore, regulation of the metabolism of intestinal microorganisms is necessary for the normal physiological activities of the body (Matsumoto et al., 2012).

Moreover, the intestinal microbial community affects the circadian rhythm of the body by altering the population structure and functional activities (Koh et al., 2018). For example, the composition of the intestinal flora changes with the transition between day and night in mice. More than 15% of the total bacterial abundance in mice changes periodically, such as the abundances of *Clostridium, Lactobacillus* and *Bacteroides* species, accounting for approximately 60% of the total intestinal bacteria (Wang et al., 2018). Fluctuations in the intestinal flora affect brain activity via the two-way connection of the gut-brain axis, thereby directly or indirectly affecting the physiological activities regulated by the central nervous system of the host (Golombek & Rosenstein, 2010; Mu, Yang & Zhu, 2016). Therefore, there may be some associations between the intestinal microbial community and the host biological clock (Mu, Yang & Zhu, 2016; Ryan et al., 2017). Regular movement of intestinal microorganisms may affect the circadian rhythm of the host by exposing intestinal tissues to different microorganisms or certain metabolites (Thaiss et al., 2016).

Birds are sensitive to illumination, and illumination can regulate biological rhythm by directly affecting vision and pineal glands. As one of the most important environmental conditions in the broiler rearing process, illumination significantly impacts the production performance of broilers. In the daily feeding of Arbor Acre (AA) broilers, to promote rapid increases in broiler weight, the illumination time can be as long as 23 h per day (Abbas et al., 2008). In addition, the microbial community structure in the intestinal tract of broilers has an important influence on the weight gain of broilers (Herrero-Encinas et al., 2020). Melatonin ameliorates lipid metabolic disorder induced by light rhythm disruption by regulating the circadian rhythms of the GI tract and the liver. Melatonin improves the diversity and richness of the microbiota (Hong et al., 2020). However, the mechanisms by which the circadian rhythm system affects the structure and metabolic activity of the intestinal microflora in broilers remain unclear. An analysis of the molecular mechanism by which light affects the performance of broiler chickens through the intestinal microbial community structure is very meaningful for the healthy breeding of poultry. Hence, in this study, broiler chickens were fed under different light cycles to detect differences in production performance and intestinal microbial community structure. In addition, serum hormone levels and the expression of related genes in tissues was measured. We tried to explain how light affects feed utilization through broiler intestinal bacterial communities. We speculate that if light can affect the absorption and utilization of nutrients in broilers through changes in the microbial community structure, the FCR of broilers can be further improved through the synergistic effect of probiotics and light system.

## Materials and Methods

### Animal management and light program

Twenty-day-old, 0.5 ± 0.05 kg AA broilers were purchased from Doyoo (Henan) Industrial Co., Ltd. Five hundred forty 20-day-old AA broilers (equal numbers of males and females) were reared under a short photoperiod (1L:23D) (A groups), a long photoperiod (23L:1D) (B groups), and normal photoperiod (16L:8D) (C groups). The light intensity was 15 lx. Each group contained three replicates, and each replicate contained 60 individuals. Conventional procedures were used for feeding the broilers (feeding conditions in the Table S1). The chickens were provided free access to food and water and were not replaced after blood sampling. The study was conducted in accordance with the Declaration of Helsinki and with the Guide for Care and Use of Laboratory Animals as adopted and promulgated by the United National Institutes of Health. The Institutional Animal Care and Use Committee of Shaanxi Normal University provided full approval for this research (SNNU-IACUC-EAC-008-2010).

When testing the serum hormone levels, six chickens are needed for each replicate group, and 18 chickens are required for each experiment group. A total of 108 chickens are required for each test under light and dark. When detecting changes in the expression levels of the corresponding enzymes, in order to avoid the stimulation of blood sampling stress, another batch of chickens, also 108 chickens were used. For 16S rDNA sequences, six chickens are needed for each replicate group, and 18 chickens are required for each experiment group, and a total of 54 chickens are required. A total of 270 chickens were used in the final experiment.

### Broilers feed conversion ratio

During the feeding period, each replicate of broilers was weighed every week, and the feed consumption was recorded. After the test, the weekly weight gain, body weight and total feed conversion ratio (FCR) of broilers were calculated.

### 16S rDNA sequences

Four hundred broilers were slaughtered at 42 days old. Six broilers from each replicate group were randomly selected for collecting the contents of the middle of the small intestine after slaughter using CO_2_ gas. All samples were immediately harvested and frozen in liquid nitrogen. The frozen luminal samples were stored in a freezer at −80 °C for subsequent use. Six chickens were dissected from each replicate, and five g of the small-intestinal content was collected. Total DNA was extracted as described in the literature (Hong et al., 2020). The extracted DNA samples from the same replicate group were mixed together and sent to Biomarker Technologies, and the V3-V4 region of the 16S rDNA gene was sequenced using an Illumina HiSeq 2500 instrument. Using UCLUST in QIIME (version 1.8.0) software, tags were clustered at the 97% similarity level to obtain operational taxonomic units (OTUs). The OTUs were annotated based on the Silva (bacteria) and UNITE (fungi) taxonomic databases.

### Prediction and analysis of the functional COGs based on 16S rDNA sequencing data

Using Phylogenetic Investigation of Communities by Reconstruction of Unobserved States (PICRUSt) software, the functional genomic compositions of samples were inferred by comparing the species composition data obtained by 16S sequencing and analyzing the functional differences among different samples or groups. Because the 16S rDNA gene differs among different species, the OTU table was standardized. Clusters of Orthologous Groups of proteins (COG) families could be identified via Greengene IDs corresponding to the OTUs, and COG abundances could be computed. The pathway information for the OTU abundances could be obtained from the COGs database.

For the abundances of functional categories at the genus level. The *G* test (for large samples, where the number of functional genes annotated was more than 20) and Fisher’s test (for small samples, where the number of functional genes annotated was less than 20) were used to test significant differences between two samples. Two-tailed *t*-tests were performed between different groups, and the *P*-value threshold was 0.05 (<0.05 indicates significance).

### Detection of melatonin, insulin and glucagon

At 42 days old, six broilers were randomly selected from each replicate group used for detection of melatonin, insulin and glucagon at each sampling point, respectively. The sampling point under lighting conditions is 8 h after turning on the light (short photoperiod group is 1 h after turning on the light). The sampling point in dark conditions is 8 h after turning off the light (long photoperiod group is 1 h after turning off the light). These broilers are not the same one used for 16S rDNA sequences. Then, two ml of blood sample was drawn from the wing vein of each broiler for serum preparation, and the sample was stored at −20 °C. The levels of melatonin, insulin and glucagon were determined according to the instructions for the ELISA kit (IBL, Hamburg, Germany) (Bagci et al., 2017; Hong et al., 2020).

### Real-time PCR

Ten-gram pancreas samples were extracted from six broilers, and 20 ml of water was added to each sample. RNA was extracted according to the instructions for the reagent, and reverse transcription was performed for preparation of cDNA. Reagent kits for RNA extraction and cDNA preparation were purchased from Omega, USA. The cDNA obtained was stored at −80 °C until further use.

Reagents for real-time PCR were purchased from ABI, USA. The primer sequences of the target genes *preproinsulin, proglucagon* and *gapdh* were designed by using ABI Primer Express (Table 1) (Hang et al., 2005), and the real-time PCR assays were performed with an ABI 7500 Fast RT-PCR instrument (Applied Biosystems, Inc., ABI, Foster City, CA, USA). Primer gradients, temperature gradients, and standard dilution curves were determined for each primer pair. The reaction conditions for real-time PCR were as follows: predenaturation at 95 °C for 10 min, followed by 40 cycles of denaturation at 95 °C for 15 s, annealing at 62 °C, and extension for 30 s. The reaction volume was 10 µL, and the reaction contained forward and reverse primers, ABI qPCR Master Mix (ABI, Foster City, CA, USA) and cDNA. Each sample was prepared in triplicate. The *gapdh* gene was used as an internal reference, and the relative expression of all genes was calculated using the 2^(−ΔΔC(T))^ method (Livak & Schmittgen, 2001).

**Table 1:** Primer sequences for real-time quantitative RT-PCR assays of target and reference genes.

Target gene	Prime name	Sequence 5′–3′
*preproinsulin*	PP-F	TCCGATCACTGCCTCTTC
	PP-R	GCTGCTCGACATCCCGTC
*proglucagon*	PG-F	CTTCCCAGTCTGAACC
	PG-R	TGTCCTCCTGTCCTTG
*gapdh*	GAPDH-F	GCCCAGAACATCATCCCA
	GAPDH-R	CGGCAGGTCAGGTCAACA

### Statistical analysis

All data were analyzed using one-way ANOVA with SPSS 20.0 (SPSS Inc., Chicago, IL, USA). All data are presented as averages with three replicates for each group. *P* < 0.05 was defined as a statistically significant difference.

## Results

### Effects of different photoperiods on the growth and feed conversion ratio of broilers

Broilers were housed under 1L/23D, 23L/1D and 16L/8D photoperiod periods for 3 weeks. During the experimental period, each group of broilers grew well and had no abnormal behavior. Infrared photography showed that the feeding periods of broilers in the 23L/1D and 16L/8D groups were the same, while the feeding periods of broilers in the 1L/23D group were approximately 10% longer than those in the two other groups (Fig. S1). During the 3-week experimental period, the feed intake of each group of broilers was calculated. The results showed that the average feed intake of each broiler in the 1L/23D group was 3.64 kg/21 d, and the energy intake was 47 MJ. The average feed intake of each broiler in the 23L/1D group was 3.02 kg/21 d, and the energy intake was 39 MJ. The average feed intake of each broiler in the 16L/8D group was 2.82 kg/21 d and the energy intake were 36.5 MJ. The body weights and overall feed-to-meat ratios of the broilers were tested. The results showed that there was no significant difference in body weight among the three groups (*P* = 0.431), but the FCR of the 1L/23D group was significantly higher (20%) than those of the other two groups (Table 2) (*P* = 0.026). This result indicated that the 1L/23D photoperiod pattern negatively affected the absorption and utilization of nutrients in the broilers.

**Table 2:** Weights, feed conversion ratios, feed consumption and weight gain of broilers under different photoperiods (mean ± SD, *n* = 6).

Group	Photoperiod	Feed-to-meat ratio	Live body weight (at 42 days) in kg	Average feed intake (22 days) in Kg	Weight gain (22 days) in Kg
Short photoperiods	1L:23D	2.23 ± 0.22^a^	2.185 ± 0.42	3.64 ± 0.39^a^	1.632 ± 0.21
Long photoperiods	23L:1D	1.81 ± 0.16^b^	2.215 ± 0.32	3.02 ± 0.26^b^	1.686 ± 0.36
Normal photoperiods	16L:8D	1.76 ± 0.14^b^	2.208 ± 0.28	2.86 ± 0.31^b^	1.649 ± 0.29

**Note:**

^a,b^Means not sharing the same superscript letter across each column differ at *P* ≤ 0.05.

### 16S rDNA sequencing and analysis

**Figure 1:**
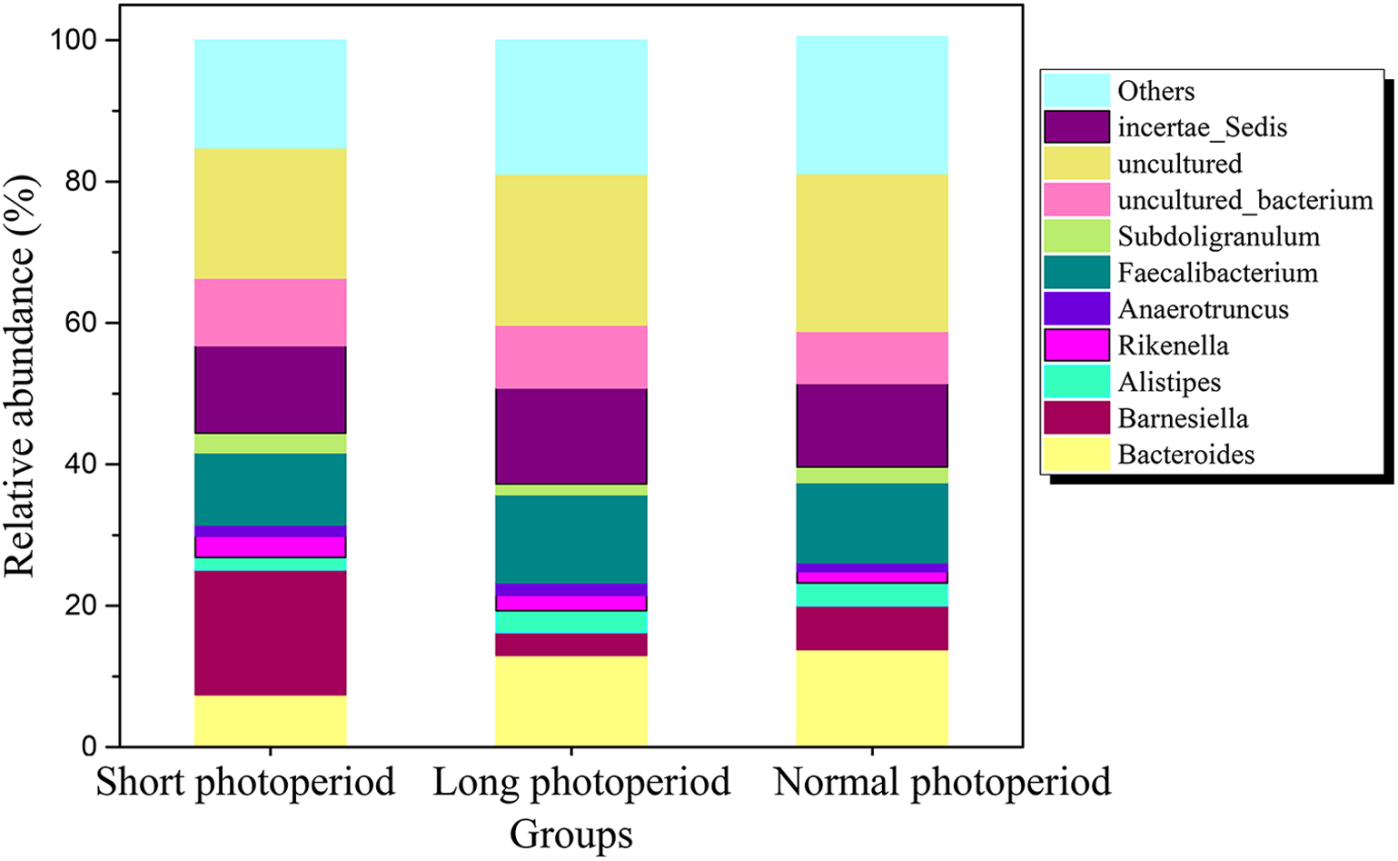
Distribution of bacterial communities at the genus level in the small intestine of AA broilers under different photoperiods. Short photoperiods:1L:23D, long photoperiods:23L:1D, normal photoperiods:16L:8D; *n* = 6.

**Figure 2:**
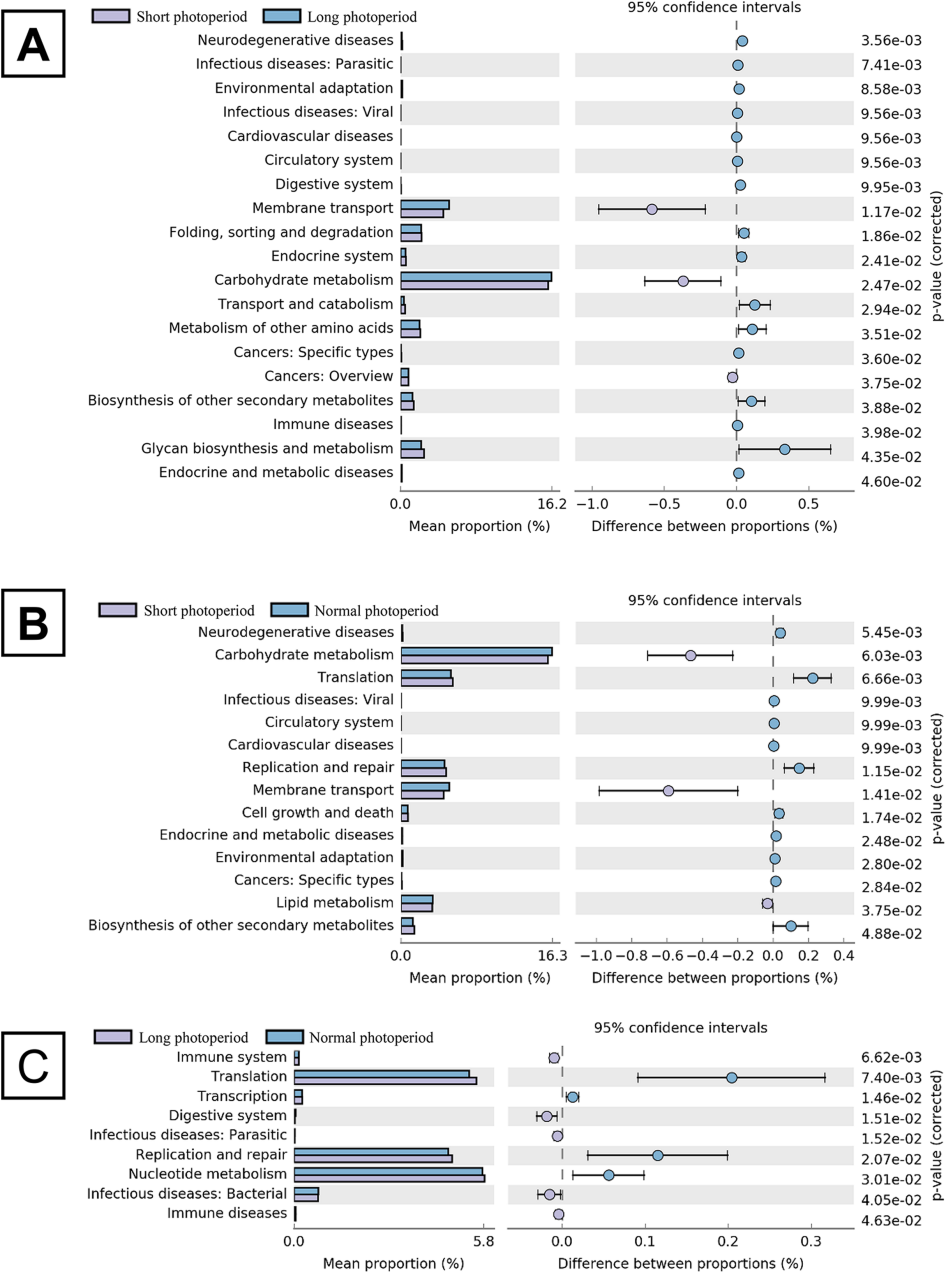
Statistical analysis of COG function of intestinal bacterial community in broilers under different photoperiods. The left panel shows the abundance ratio of the different functions in the two groups. The middle shows the difference in functional abundance in the 95% confidence interval, and the rightmost value is the *P*-value. The picture (A) shows the comparison of groups A and B. The picture (B) shows the comparison of groups A and C. The picture (C) shows the comparison of groups B and C. Short photoperiods:1L:23D, long photoperiods:23L:1D, normal photoperiods:16L:8D; *n* = 6.

After the broilers were fed for 3 weeks, three chickens were randomly selected from each repeat group to collect intestinal contents. Total DNA was extracted from the intestinal contents, and the V3-V4 region of the 16S rDNA gene was amplified by PCR for sequencing. Sixty thousand valid sequences were obtained for each test sample, covering a wide range of regions and a high abundance of sequences for further analysis. The identified sequences were subjected to a BLAST analysis against the NCBI microbial 16S rDNA database to determine the compositions of the bacterial community in the small intestines of the broilers. The BLAST results showed that the abundance of *Barnesiella* in the small intestines of the broilers in the 1L/23D group was three to six times higher than that of the broilers in the other two experimental groups, constituting the highest (20%) percentage of the total bacteria. The abundances of *Bacteroides* and *Alistipes* in the 1L/23D group were less than half of those in the other two experimental groups, accounting for less than 5% of the total bacterial content (Fig. 1). The COG database was used to analyze the possible metabolic functions of the bacteria corresponding to the identified sequences. The results showed that the abundances of bacterial genes involved in glycometabolism and membrane transport in the small intestines of broilers under 16L/8D and 23L/1D were greater than those in the intestines of broilers subjected to only 1 h of illumination daily (Fig. 2).

### Effects of different photoperiods on the serum levels of melatonin, insulin and glucagon

**Figure 3:**
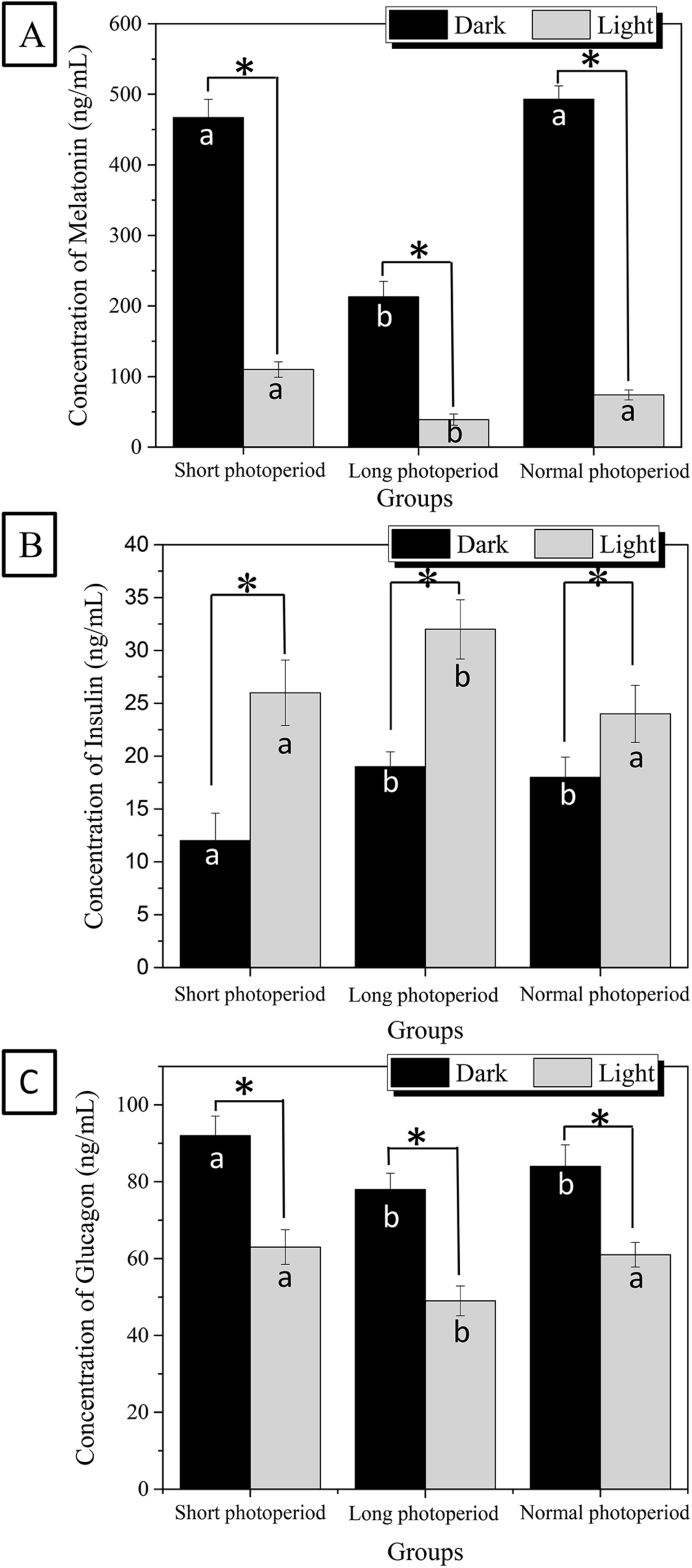
Differences of melatonin (A), insulin (B) and glucagon (C) concentrations in the broilers serum under different photoperiods. Short photoperiods:1L:23D, long photoperiods:23L:1D, normal photoperiods:16L:8D; *n* = 6. *Means significant difference within the group, *P* < 0.05; a, b in the column represent significant difference between groups under dark/light condition, *P* < 0.05, the columns carry different letters are significantly differed while columns carry same letters are insignificantly differed.

The effect of illumination on the body is mainly reflected in the regulation of melatonin synthesis. Insulin and glucagon are critical hormones that regulate fat synthesis and have important effects on fat storage in broilers. To further elucidate the molecular mechanism by which light affects the growth and development of broilers, the serum levels of melatonin, insulin and glucagon were measured. As shown in Fig. 3, the concentration of melatonin in the sera of broilers raised under the 1L/23D period reached 470 ng/mL under dark conditions, which was approximately four times higher than the concentration observed under illuminated conditions. The concentration of melatonin in the sera of broilers raised under the 23L/1D period was only 210 ng/mL under dark conditions and 27 ng/mL under illuminated conditions (Fig. 3). The highest concentration of serum melatonin that was reached under 16L/8D photoperiod was similar to the concentration observed in broilers raised under 1L/23D in dark conditions, and the lowest concentration of melatonin under illuminated conditions was slightly lower than that observed under the 1L/23D period.

The changes in serum insulin and glucagon levels also exhibited significant rhythmicity. The concentrations of both serum insulin and glucagon decreased in each group under dark conditions and increased under illuminated conditions. The 27 ng/ml of highest value of insulin concentration in broiler serum appeared at the illuminated conditions of 23L/1D period, which was approximately 20% higher than the concentrations observed for the other two experimental groups under illuminated conditions. The 90 ng/ml of highest value of glucagon concentration in broiler serum appeared at the dark conditions of 1L/23D period, which was approximately 12% higher than the concentrations observed for the other two groups under dark conditions (Fig. 3). This finding indicates that the photoperiod has a specific effect on the synthesis of insulin and glucagon. As the photoperiod changed, the levels of insulin and glucagon in the serum also showed a significant rhythm. The experimental results are consistent with the fat accumulation kinetics of more fat consumption occurring during the day and more fat accumulation occurring at night. It is also consistent with the role in fat metabolism of insulin and glucagon.

### Effects of different photoperiods on the transcriptional levels of the proinsulin and glucagon genes in the pancreas

To determine whether changes in the photoperiod affect the synthesis of insulin and glucagon, the transcriptional levels of the proinsulin and glucagon genes were examined by real-time PCR (Livak & Schmittgen, 2001; Gonzalez-Arto et al., 2016).

**Figure 4:**
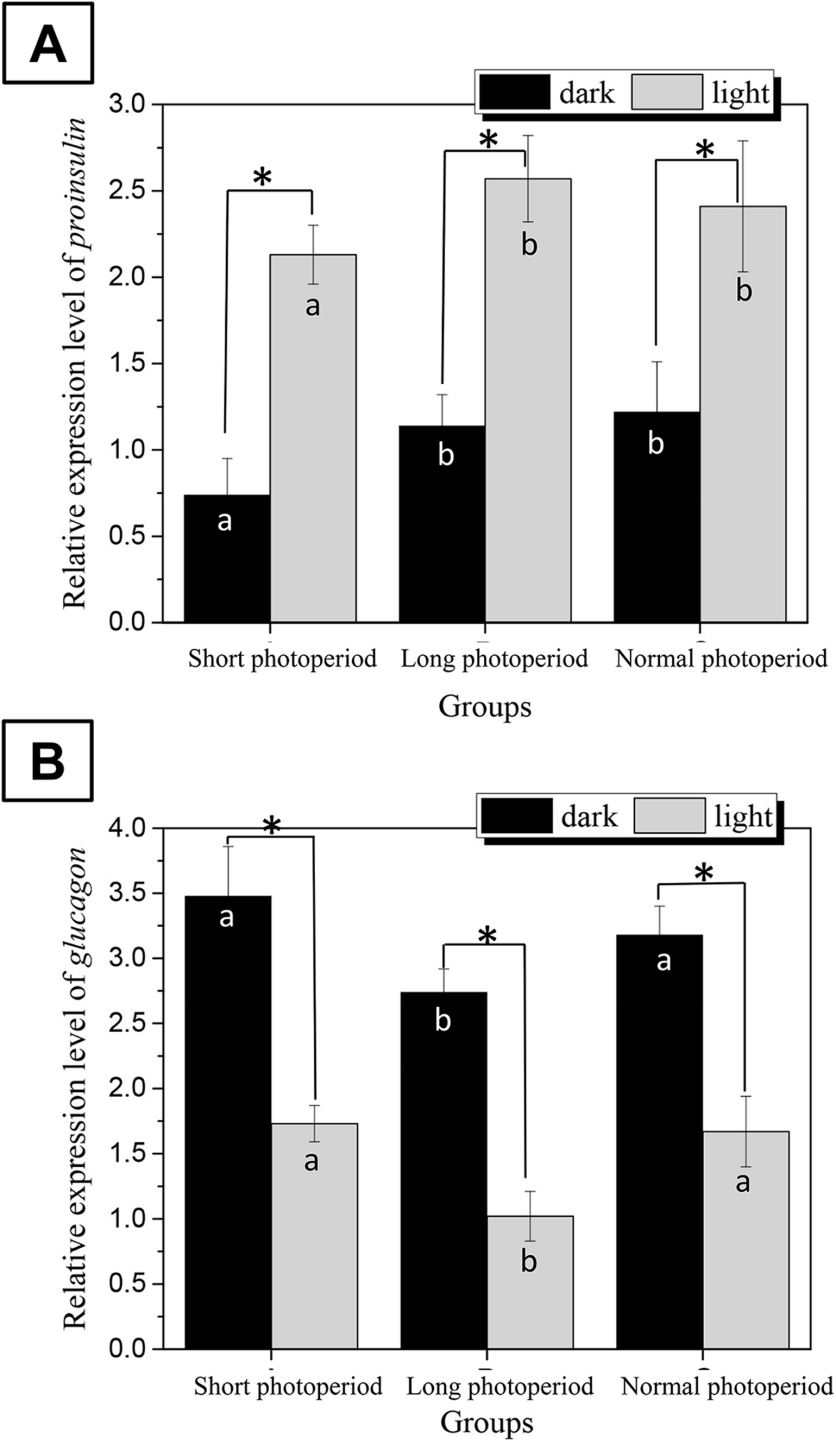
Differences in the relative expression levels of *proinsulin* (A) and *glucagon* (B) in the broilers pancreas under different photoperiods. *Proinsulin* and *glucagon* are the genes encoding the key enzymes for the synthesis of insulin and glucagon in broilers, respectively. The gapdh gene was used as the internal reference, and the relative expression of all genes was calculated using the 2^(−ΔΔC(T))^ method. Short photoperiods:1L:23D, long photoperiods:23L:1D, normal photoperiods:16L:8D; *n* = 6. *Means significant difference within the group, *P* < 0.05; a, b in the column represent significant difference between groups under dark/light condition, *P* < 0.05, the columns carry different letters are significantly differed while columns carry same letters are insignificantly differed.

The changes in the transcriptional levels of the proinsulin and glucagon genes were consistent with the changes in serum insulin and glucagon levels. The abundance of proinsulin mRNA in the pancreas of broilers subjected to a 23L/1D period was 2.6 times higher than that under dark conditions and two times higher than that under illuminated conditions in the other two experimental groups. The abundance of glucagon mRNA in the pancreas of broilers subjected to a 1L/23D period was 2.2 times higher than that under illuminated conditions and 1.6 times higher than that under dark conditions in the other two experimental groups (Fig. 4). This finding indicates that changes in the photoperiod affect the synthesis of insulin and glucagon.

## Discussion

### Effect of illumination on broiler feeding

Illumination is an important factor affecting the circadian rhythms of animals. Long-term changes in the light cycle can change the circadian rhythm systems of animals and can affect their reproduction, metabolism, immunity and nervous systems (Kim et al., 2018; Org et al., 2015; Schwean-Lardner, Fancher & Classen, 2012). The conventional belief is that chickens do not eat under dark conditions, but in this study, the chickens exhibited increased ingestion of food under prolonged dark conditions, which may have been a result of the chickens being kept in a restricted space and not needing to seek food, as the food was in close proximity to the chickens in this study. Under prolonged dark conditions, chickens increase food consumption to meet the energy needs of their bodies.

### Illumination affects bacterial community structure and function

Changes in illumination time also alter the microbial community structure in animals (Voigt et al., 2016; Wu et al., 2018; Liang & FitzGerald, 2017). The gut microbial community of animals is closely associated with metabolism, immunity and nutrient absorption and utilization systems (Thaiss et al., 2016). Intestinal microorganisms can digest organic compounds that are difficult for animals to directly absorb and utilize, converting these compounds into small molecules for absorption in the small intestine by methods, such as decomposing polysaccharides to monosaccharides and oligosaccharides (Mikkelsen et al., 2015). Intestinal microbes can also increase the sugar transport ability of the intestinal mucosa. Therefore, a normal microbial community is very important for the maintenance of normal nutrient absorption in hosts (Liang & FitzGerald, 2017). During the growth of newly hatched chicks, different light regimes cause changes in the circadian rhythm of the clock gene, which in turn changes the structure of intestinal microbes. However, it is unclear how the light cycle affects broiler gut microbiota during fattening (Hieke, Hubert & Athrey, 2019).

### Bacterial community structure affects insulin synthesis

Insulin and glucagon are the main hormones that regulate fat synthesis. Insulin promotes fat synthesis, and glucagon inhibits fat synthesis. Under prolonged illumination, the synthesis of melatonin increases, inhibiting the synthesis of insulin, promoting the synthesis of glucagon, and facilitating the synthesis of fat. Intestinal microbial communities can also affect the endocrine systems of animals via the intestine-brain axis. For example, intestinal microorganisms can alter the synthesis and secretion of insulin and glucagon, which is closely related to type II diabetes mellitus in animals (Wei et al., 2018). The intestinal microbial community also has its own circadian rhythm system, which corresponds to the host rhythm system and maintains the normal life cycle of the organism. Instability in the circadian rhythm system can cause many systemic disorders in the endocrine system, metabolism and immunity of the host (Khalyfa et al., 2017).

Birds are sensitive to light, and light can directly affect the pineal glands of chickens and change the intrinsic circadian rhythm systems of these birds. The results of this study show that the long photoperiod caused changes not only in the levels of melatonin, an important circadian rhythm factor, but also, in microbial community structure and in the levels of a series of hormones. Changes in the circadian rhythm lead to changes in the synthesis and secretion of melatonin. As an important endocrine hormone, melatonin affects the levels of insulin and glucagon (Peschke, Bahr & Muhlbauer, 2013; Sharma et al., 2015). Insulin and glucagon are synthesized and secreted by beta cells and alpha cells in islets. In pancreatic beta cells, the proinsulin gene is the key gene for the synthesis of mature insulin under the action of a series of proteolytic enzymes. The glucagon gene is the key gene involved in the synthesis of glucagon. The glucagon gene in islet alpha cells synthesizes proglucagon. An increase in the melatonin content can enhance the syntheses of insulin and glucagon. This change is beneficial for the synthesis of fat. Insulin breaks down glucose for fat synthesis and lowersblood sugar levels.

The prolonged illumination time increased the abundances of *Bacteroides* and *Alistipes* species, increased the hydrolysis of polysaccharides to monosaccharides and oligosaccharides, and increased the sugar transport capacity of intestinal mucosal cells (Wei et al., 2018; Yin et al., 2018; Agans et al., 2018). Simultaneously, prolongation of the illumination time reduced the abundances of *Barnesiella* bacteria in the chicken intestine; these bacteria can synthesize short-chain fatty acids from sugar, thus reducing the rate of sugar utilization in the chicken intestine (Wei et al., 2018). Short- and medium-chain 1-monoglycerides positively modulated the fish intestinal microbiota by increasing the number of beneficial lactic acid bacteria, namely, Lactobacillus, and reducing Gammaproteobacteria, which include several potential pathogenic bacteria (Rimoldi et al., 2018). Monosaccharides and oligosaccharides are transported into small intestinal mucosal cells by the phosphotransferase system and SGLT-1 transporter protein, and fat is synthesized in liver and stored in the liver and adipose tissue, thus increasing feed utilization (Thaiss et al., 2016). Therefore, under prolonged illumination, the utilization of sugars in the host for fat synthesis increases, which reduces the FCR. Hence, an appropriate increase in illumination time reduces the feed-to-meat ratio during broiler feeding. To the best of our knowledge, this study is the first to systematically explain the molecular mechanism underlying the effect of light on the feed-to-meat ratio of broilers in terms of circadian rhythm and changes in intestinal microbial community structure.

## Conclusion

Light can affect the concentration of melatonin in blood, change the circadian rhythm systems of chickens, and affect the small intestinal bacterial community. Prolongation of illumination time can reduce the abundances of *Barnesiella* bacteria and increase the abundances of *Bacteroides* and *Alistipes* species. The changes in bacterial community structure observed under long-term illumination enhanced the glycometabolism and membrane transport ability of the intestinal bacteria. Simultaneously, the changes in the intestinal microflora increased the synthesis of insulin and decreased the synthesis of glucagon. These variations were conducive to the synthesis of fat in liver and adipose tissue. In conclusion, the increase in fat and the increase in membrane transport capacity led to the FCR of the broilers decreasing under prolonged illumination and increasing under dark conditions. This study explains the mechanism by which prolonged light exposure reduces the broiler FCR in terms of a synergistic light-melatonin-intestinal flora-insulin effect.

## Supplemental Information

10.7717/peerj.9638/supp-1 Supplemental Information 1 Dietary formula and nutrient level of corn-bean meal type for broiler chickens (dry basis, %).

10.7717/peerj.9638/supp-2 Supplemental Information 2 Feeding time of broilers under different photoperiods. Short photoperiods:1L:23D, long photoperiods:23L:1D, norma photoperiods:16L:8D; *n* = 6. *Represents significant differences in dark and light, *P* < 0.05.

## Additional Information and Declarations

### DNA Deposition

The following information was supplied regarding the deposition of DNA sequences: Data is available at GenBank: SAMN14133017, SAMN14133018, SAMN14133019, SAMN14133020, SAMN14133021, SAMN14133022, SAMN14133023, SAMN14133024, SAMN14133025, and PRJNA607432.

### Data Availability

The following information was supplied regarding data availability: Data is available at Figshare: wang, yongfen (2020): Molecular Mechanism Underlying the Effect of Illumination Time on the Growth Performance of Broilers via Changes in the Intestinal Bacterial Community. figshare. Dataset. DOI 10.6084/m9.figshare.11860650.v1.

## References

[ref-1] Abbas AO, El-Dein AKA, Desoky AA, Galal AAM (2008). The effects of photoperiod programs on broiler chicken performance and immune response. International Journal of Poultry Science.

[ref-2] Agans R, Gordon A, Kramer DL, Perez-Burillo S, Rufian-Henares JA, Paliy O (2018). Dietary fatty acids sustain the growth of human gut microbiota. Applied and Environmental Microbiology.

[ref-3] Bagci S, Altuntas Ö, Katzer D, Berg C, Willruth A, Reutter H, Bartmann P, Müller A, Zur B (2017). Evaluation of two commercially available ELISA kits for the determination of melatonin concentrations in amniotic fluid throughout pregnancy. Annals of Clinical Biochemistry.

[ref-4] Claus SP, Ellero SL, Berger B, Krause L, Bruttin A, Molina J, Paris A, Want EJ, De Waziers I, Cloarec O, Richards SE, Wang Y, Dumas ME, Ross A, Rezzi S, Kochhar S, Van Bladeren P, Lindon JC, Holmes E, Nicholson JK (2011). Colonization-induced host-gut microbial metabolic interaction. mBio.

[ref-5] Golombek DA, Rosenstein RE (2010). Physiology of circadian entrainment. Physiological Reviews.

[ref-6] Gonzalez-Arto M, Hamilton TR, Gallego M, Gaspar-Torrubia E, Aguilar D, Serrano-Blesa E, Abecia JA, Pérez-Pé R, Muiño-Blanco T, Cebrián-Pérez JA, Casao A (2016). Evidence of melatonin synthesis in the ram reproductive tract. Andrology.

[ref-7] Hang XM, Power D, Flik G, Balment RJ (2005). Measurement of PTHrP, PTHR1, and CaSR expression levels in tissues of sea bream (*Sparus aurata*) using quantitative PCR. Annals of the New York Academy of Sciences.

[ref-8] Herrero-Encinas J, Blanch M, Pastor JJ, Mereu A, Ipharraguerre IR, Menoyo D (2020). Effects of a bioactive olive pomace extract from *Olea europaea* on growth performance, gutfunction, and intestinal microbiota in broiler chickens. Poultry Science.

[ref-35] Hieke AC, Hubert SM, Athrey G (2019). Circadian disruption and divergent microbiota acquisition under extended photoperiod regimens in chicken. PeerJ.

[ref-9] Hong F, Pan S, Xu P, Xue T, Wang J, Guo Y, Jia L, Qiao X, Li L, Zhai Y (2020). Melatonin orchestrates lipid homeostasis through the hepatointestinal circadian clock and microbiota during constant light exposure. Cells.

[ref-10] Khalyfa A, Poroyko VA, Qiao Z, Gileles-Hillel A, Khalyfa AA, Akbarpour M, Almendros I, Farre R, Gozal D (2017). Exosomes and metabolic function in mice exposed to alternating dark-light cycles mimicking night shift work schedules. Frontiers in Physiology.

[ref-11] Kim DH, Jeong D, Kim H, Seo KH (2018). Modern perspectives on the health benefits of kefir in next generation sequencing era: improvement of the host gut microbiota. Critical Reviews in Food Science and Nutrition.

[ref-12] Koh A, Molinaro A, Stahlman M, Khan MT, Schmidt C, Manneras-Holm L, Wu H, Carreras A, Jeong H, Olofsson LE, Bergh PO, Gerdes V, Hartstra A, De Brauw M, Perkins R, Nieuwdorp M, Bergstrom G, Backhed F (2018). Microbially produced imidazole propionate impairs insulin signaling through mTORC1. Cell.

[ref-13] Leone V, Gibbons SM, Martinez K, Hutchison AL, Huang EY, Cham CM, Pierre JF, Heneghan AF, Nadimpalli A, Hubert N, Zale E, Wang Y, Huang Y, Theriault B, Dinner AR, Musch MW, Kudsk KA, Prendergast BJ, Gilbert JA, Chang EB (2015). Effects of diurnal variation of gut microbes and high-fat feeding on host circadian clock function and metabolism. Cell Host & Microbe.

[ref-14] Liang X, FitzGerald GA (2017). Timing the microbes: the circadian rhythm of the gut microbiome. Journal of Biological Rhythms.

[ref-15] Livak KJ, Schmittgen TD (2001). Analysis of relative gene expression data using real-time quantitative PCR and the 2^−ΔΔCT^ method. Methods.

[ref-16] Matsumoto M, Kibe R, Ooga T, Aiba Y, Kurihara S, Sawaki E, Koga Y, Benno Y (2012). Impact of intestinal microbiota on intestinal luminal metabolome. Scientific Reports.

[ref-17] Mikkelsen KH, Frost M, Bahl MI, Licht TR, Jensen US, Rosenberg J, Pedersen O, Hansen T, Rehfeld JF, Holst JJ, Vilsboll T, Knop FK (2015). Effect of antibiotics on gut microbiota, gut hormones and glucose metabolism. PLOS ONE.

[ref-18] Mohawk JA, Green CB, Takahashi JS (2012). Central and peripheral circadian clocks in mammals. Annual Review of Neuroscience.

[ref-19] Mu C, Yang Y, Zhu W (2016). Gut microbiota: the brain peacekeeper. Frontiers in Microbiology.

[ref-20] Nicholson JK, Holmes E, Kinross J, Burcelin R, Gibson G, Jia W, Pettersson S (2012). Host-gut microbiota metabolic interactions. Science.

[ref-21] Org E, Parks BW, Joo JW, Emert B, Schwartzman W, Kang EY, Mehrabian M, Pan C, Knight R, Gunsalus R, Drake TA, Eskin E, Lusis AJ (2015). Genetic and environmental control of host-gut microbiota interactions. Genome Research.

[ref-22] Peschke E, Bahr I, Muhlbauer E (2013). Melatonin and pancreatic islets: interrelationships between melatonin, insulin and glucagon. International Journal of Molecular Sciences.

[ref-23] Rimoldi S, Gliozheni E, Ascione C, Gini E, Terova G (2018). Effect of a specific composition of short- and medium-chain fatty acid 1-Monoglycerides on growth performances and gut microbiota of gilthead sea bream (*Sparus aurata*). PeerJ.

[ref-24] Ryan PM, London LE, Bjorndahl TC, Mandal R, Murphy K, Fitzgerald GF, Shanahan F, Ross RP, Wishart DS, Caplice NM, Stanton C (2017). Microbiome and metabolome modifying effects of several cardiovascular disease interventions in apo-E−/− mice. Microbiome.

[ref-25] Schwean-Lardner K, Fancher BI, Classen HL (2012). Impact of daylength on the productivity of two commercial broiler strains. British Poultry Science.

[ref-26] Sharma S, Singh H, Ahmad N, Mishra P, Tiwari A (2015). The role of melatonin in diabetes: therapeutic implications. Archives of Endocrinology and Metabolism.

[ref-27] Thaiss CA, Levy M, Korem T, Dohnalova L, Shapiro H, Jaitin DA, David E, Winter DR, Gury-BenAri M, Tatirovsky E, Tuganbaev T, Federici S, Zmora N, Zeevi D, Dori-Bachash M, Pevsner-Fischer M, Kartvelishvily E, Brandis A, Harmelin A, Shibolet O, Halpern Z, Honda K, Amit I, Segal E, Elinav E (2016). Microbiota diurnal rhythmicity programs host transcriptome oscillations. Cell.

[ref-28] Van der Vinne V, Burgos BM, Harrington ME, Weaver DR (2020). Deconstructing circadian disruption: assessing the contribution of reduced peripheral oscillator amplitude on obesity and glucose intolerance in mice. Journal of Pineal Research.

[ref-29] Voigt RM, Forsyth CB, Green SJ, Engen PA, Keshavarzian A (2016). Circadian rhythm and the gut microbiome. International Review of Neurobiology.

[ref-30] Wang Y, Kuang Z, Yu X, Ruhn KA, Kubo M, Hooper LV (2017). The intestinal microbiota regulates body composition through NFIL3 and the circadian clock. Science.

[ref-31] Wang J, Nesengani LT, Gong Y, Yang Y, Lu W (2018). 16S rRNA gene sequencing reveals effects of photoperiod on cecal microbiota of broiler roosters. PeerJ.

[ref-32] Wei X, Tao J, Xiao S, Jiang S, Shang E, Zhu Z, Qian D, Duan J (2018). Xiexin Tang improves the symptom of type 2 diabetic rats by modulation of the gut microbiota. Scientific Reports.

[ref-33] Wu G, Tang W, He Y, Hu J, Gong S, He Z, Wei G, Lv L, Jiang Y, Zhou H, Chen P (2018). Light exposure influences the diurnal oscillation of gut microbiota in mice. Biochemical and Biophysical Research Communications.

[ref-34] Yin J, Li Y, Han H, Chen S, Gao J, Liu G, Wu X, Deng J, Yu Q, Huang X, Fang R, Li T, Reiter RJ, Zhang D, Zhu C, Zhu G, Ren W, Yin Y (2018). Melatonin reprogramming of gut microbiota improves lipid dysmetabolism in high-fat diet-fed mice. Journal of Pineal Research.

