# Supplementary material for: Molecular mechanism underlying the effect of illumination time on the growth performance of broilers via changes in the intestinal bacterial community
Source: PeerJ. 2020 Aug 3;8:e9638. doi: 10.7717/peerj.9638 (PMC7409780; doi:10.7717/peerj.9638)
Supplement: Supplemental Information 1 [file peerj-08-9638-s001.docx]

**Molecular Mechanism Underlying the Effect of Illumination Time on the Growth Performance of Broilers via Changes in the Intestinal Bacterial Community**

Yongfen Wang^1,2^, Zhen Zhang^2^, Pengkun Yang^2^, Miaorui Zhang^2^, Lei Xi^2^, Qiong Liu^3^, Jingang Li^1,^

^1^ College of Life Sciences, National Engineering Laboratory for Resource Development of Endangered Crude Drugs in Northwest China, Shaanxi Normal University, Xi’an, 710119, China;

^2^ College of Food and Biology Engineering, Henan University of Animal Husbandry and Economy, Zhengzhou, 450046, China;

^3^College of Biosystems Engineering and Food Science, Zhejiang University, Hangzhou, 310058, China;

corresponding author:

Jingang Li^1^

Email address: jingang@snnu.edu.cn

Broiler management

The test lamps are all LED lights and are provided by Hangzhou Langtuo Biotechnology Co., Ltd. The test broilers were housed in cages of the same specifications (length × width × height = 950 mm × 900 mm × 400 mm), and the other feeding management conditions were consistent.

The management of AA broiler chickens is strictly in accordance with the AA Broiler Management Manual. Fed three times a day at 07:00, 13:00 and 18:00, respectively. Broilers are free to eat and drink. The house temperature was controlled at 20 ° C by manual temperature control. The pre-test period is 3d and the test period is 21d. The diet was a full-price compound feed produced by Luoyang City Kunpeng Feed Co., Ltd.. Diets formula are presented in Table S1.

Table S1 dietary formula and nutrient level of corn-bean meal type for broiler chickens (dry basis, %)

| Ingredients | % |
| --- | --- |
| Corn | 64.00 |
| Soybean meal | 24.50 |
| CPM | 5.00 |
| Soybean oil | 2.50 |
| NaCl | 0.30 |
| CaHPO_4_ | 1.00 |
| Limestone | 1.30 |
| Zeolite | 0.40 |
| Premix | 1.00 |
| Total | 100.00 |
| Nutrient levels (%) | |
| Metabolic energy (MJ/kg) | 12.95 |
| CP | 20.10 |
| Lys | 1.02 |
| Met | 0.42 |
| Met+Cys | 0.78 |
| Ca | 0.81 |
| TP | 0.52 |
